# Adrenocortical insufficiency after bilateral adrenal hemorrhage due to anticoagulation and chronic immunothrombocytopenia

**DOI:** 10.1530/EDM-24-0034

**Published:** 2024-11-20

**Authors:** Sophie Charlotte Hintze, Felix Beuschlein

**Affiliations:** 1 Department of Endocrinology, Diabetology and Clinical Nutrition, University Hospital Zurich, Zurich, Switzerland

**Keywords:** adrenocortical insufficiency, adrenal crisis, adrenal hemorrhage, anticoagulation, chronic immune thrombocytopenia, Please select country of treatment for each patient reported, SWITZERLAND

## Abstract

**Summary:**

Adrenocortical insufficiency is defined as the clinical manifestation of chronic glucocorticoid and/or mineralocorticoid deficiency due to failure of the adrenal cortex. It may result in an adrenal crisis, which is a life-threatening disease; thus, prompt initiation of therapy with hydrocortisone is necessary. Symptoms such as hypotension, weight loss, or fatigue are not specific, which is why diagnosis is delayed in many cases. Our patient suffered from immune thrombocytopenia (ITP), an acquired thrombocytopenia caused by an autoimmune reaction against platelets and megakaryocytes. Primary ITP, in which no triggering cause can be identified, must be distinguished from secondary forms (e.g. in the context of systemic autoimmune diseases, lymphomas, or (rarely) by drugs). Patients may be asymptomatic at presentation or may present with a range of mild mucocutaneous to life-threatening bleeding. Here, we report on a 43-year-old woman who had developed adrenocortical insufficiency due to bilateral hemorrhage in the adrenal glands. Because of anticoagulation with phenprocoumon after pulmonary embolism and thrombocytopenia on the basis of ITP, the patient had an increased risk of bleeding. Due to the nonspecific and ambiguous symptoms of adrenocortical insufficiency, prompt diagnosis remains a challenge.

**Learning points:**

- Hypocortisolism or adrenal crisis with nonspecific symptoms, especially abdominal and gastrointestinal, is often misinterpreted.
- Diagnosis of adrenal insufficiency is often delayed because of the initial ambiguous presentation; physicians must be aware to avoid adrenal crisis.
- Especially in patients with several risk factors for bleeding, unusual bleeding manifestations, such as adrenal hemorrhage, must be considered.
- Immediate treatment is necessary by substituting hydrocortisone in a higher dosage, and in most cases, fludrocortisone.
- During the course of treatment, the amount of hydrocortisone can be reduced to a substitution dosage (15–25 mg/day divided into two to three doses/day). Fludrocortisone should be continued at a dosage of 0.05–0.1 mg/day, depending on blood pressure and sodium and potassium levels.
- All patients should carry a medical alert notification or a steroid emergency card. In the case of trauma, surgery, or other stressful events, hydrocortisone must be administered in higher dosages (e.g. 100 mg i.v.).

## Background

Adrenocortical insufficiency is a life-threatening condition triggered by several disorders. It can result from a primary adrenal disorder, secondary to adrenocorticotropic hormone deficiency, or by suppression of adrenocorticotropic hormone release, e.g. due to exogenous glucocorticoid medications. About 80% of primary adrenocortical insufficiency is due to autoimmune adrenalitis. Other common causes of primary adrenocortical insufficiency are infectious (e.g. tuberculosis) and neoplastic disorders. Less frequent causes are genetic diseases, adrenal hemorrhage, or iatrogenic conditions such as pharmacological side effects (1). Symptoms and signs of adrenocortical insufficiency appear when more than 90% of the cortex is destroyed. Typical symptoms of adrenocortical insufficiency are fatigue, muscle and abdominal pain, unintentional weight loss or anorexia, hypotension, and hyponatremia. In addition, in primary adrenal insufficiency, patients usually develop skin hyperpigmentation and salt craving. In many cases, the diagnosis of adrenal insufficiency is delayed, as the initial presentation is often non-specific. The therapeutic approach is to substitute glucocorticoids, typically in the form of hydrocortisone, and mineralocorticoids like fludrocortisone. The goal of therapy is adequate substitution to restore quality of life and avoid adrenal crises. Avoiding adrenal crises (e.g. caused by acute infections like gastrointestinal infections) is fundamental, as it contributes to increased mortality. In patients with primary adrenal insufficiency, the stated incidence of adrenal crisis is about 6–8/100 patients/ year with a mortality rate of 0.5/100 patient-years. To prevent adrenal crisis, it is existential to educate the patients on how to increase medication during acute illness and profound stress. In addition, all patients with adrenal insufficiency should carry a medical alert notification or a steroid emergency card. Primary adrenal insufficiency is accompanied by the substitution of a mineralocorticoid (e.g. fludrocortisone) to stabilize water and electrolyte balance (1, 2, 3).

Immune thrombocytopenia (ITP) is a rare autoimmune disease characterized by isolated thrombocytopenia. It can be diagnosed in patients with a repeated platelet count below <100 × 10^9^/L. ITP is a diagnosis of exclusion; therefore, other causes of thrombocytopenia, such as vitamin B12 deficiency, medications (e.g. Heparin), or bone marrow disorders, need to be ruled out. Primary ITP must be distinguished from secondary forms. These secondary forms may be induced, e.g. in the context of systemic autoimmune diseases, in lymphomas, or (rarely) by drugs (4). The extent of symptoms shows a wide range, from asymptomatic patients, mucocutaneous bleeding to life-threatening bleeding. Five years after diagnosis, approximately 15% of patients develop a bleeding that leads to hospital admission, which thus represents an important factor of morbidity (5). Furthermore, patients with ITP show a twofold increased risk of venous thromboembolism compared to the general population (6).

We describe a patient with adrenal hemorrhage caused by anticoagulation after pulmonary embolism and thrombocytopenia due to ITP, leading to an adrenocortical insufficiency.

## Case presentation

We report on a 43-year-old woman with several pulmonary embolisms and consecutive chronic thromboembolic pulmonary hypertension, as well as recurrent chronic ITP, who developed adrenocortical insufficiency due to bilateral hemorrhage.

The patient has a history of pulmonary embolism, with the first episode occurring in 2003. After a second event in 2009, treatment with phenprocoumon was started. Due to a subtherapeutical dosage of phenprocoumon, she developed a pulmonary embolism again in 2010, the last episode so far. In 2010, ITP was diagnosed after she developed petechiae and mucosal bleeding. Intensive diagnostic approaches, including measurement of antiphospholipid antibodies, antibodies against systemic lupus erythematodes, rheumatoid arthritis (including anti-CCP antibodies), systemic sclerosis, antibodies against synthetase, antinuclear antibodies, and anti-neutrophil cytoplasmic antibodies (ANCA), were negative. No pathological immunoglobulins or kappa/lambda paraproteins were found. Therefore, ITP appeared to be the cause of the venous thromboembolism. Since 2010, ten recurrences of ITP have occurred, which have been treated with steroids (dexamethasone), rituximab, intravenous immunoglobulin, and eltrombopag.

In the course of time, she developed pulmonary hypertension, which was diagnosed as chronic thromboembolic pulmonary hypertension. For treatment, she underwent bilateral pulmonary endarterectomy in 2017.

In addition, the patient has chronic renal insufficiency with an eGFR of 30–45 mL/min. Recurrent acute kidney injuries in the context of the above-mentioned events and hypertensive nephropathy have been postulated as potential causes.

In November 2022, the patient presented to the emergency department due to abdominal pain and fatigue. At that time, further investigations, including a CT scan, did not reveal any conclusive abdominal findings. Especially, the adrenal glands were described as normal. Likewise, sodium and potassium levels were within the normal range. C-reactive protein was elevated to 227 mg/L (reference value <5 mg/L). The cause of the complaints was postulated to be a nitrite-positive urinary tract infection, which was treated with piperacillin/tazobactam. Fourteen days later, the patient was hospitalized again for 1 week due to fatigue, anorexia, persistent abdominal pain, nausea, and vomiting. The symptoms were evaluated as gastritis. In addition, acute on chronic renal failure was evident, which was interpreted as prerenal based on the vomiting.

## Investigation

**Figure 1:**
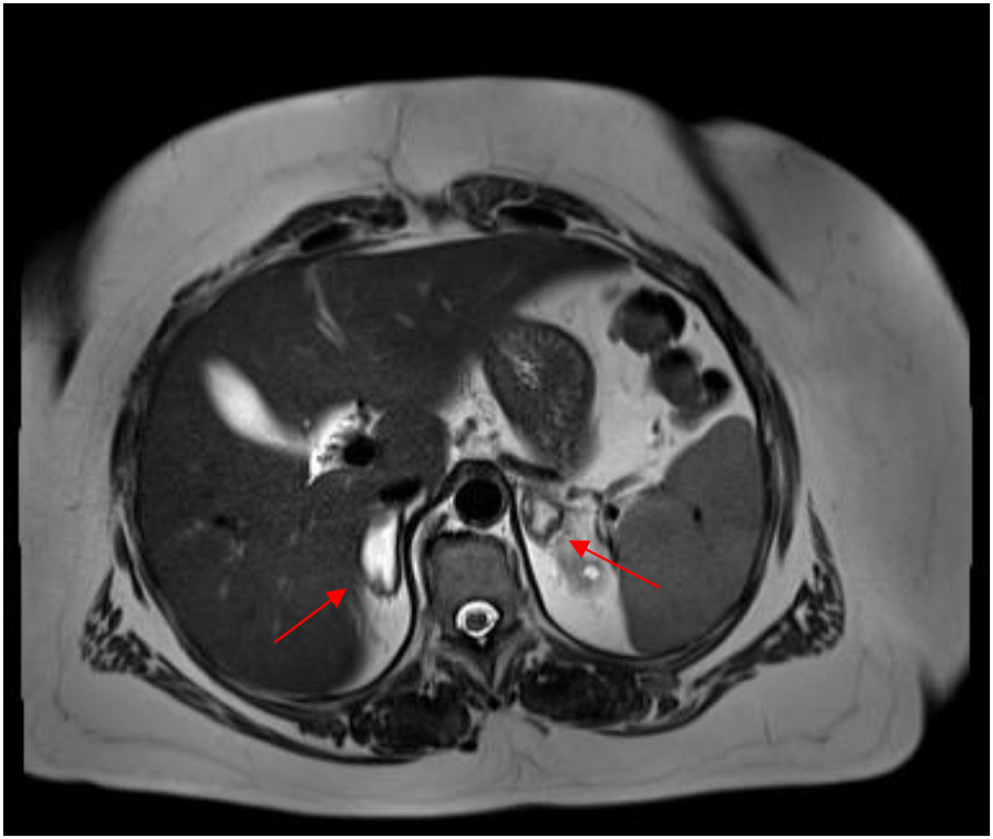
MRI abdomen in transverse plane showing bilateral hemorrhage in the adrenal glands (arrows) on a T2-weighted image.

As the above-mentioned complaints did not improve in the further course, an MRI of the abdomen was added in December 2022. This MRI showed, in contrast to the earlier CT scan, enlarged adrenal glands on both sides with markedly hyperintense signal in T2, interpreted as bilateral adrenal hemorrhage (Fig. 1). A detailed evaluation of the MRI examination did not demonstrate active bleeding and revealed that the hemorrhage in the adrenal glands must have occurred several weeks prior to the MRI examination. Given the impressive radiological findings, analysis of the basal cortisol level as well as the ACTH level was performed (Table 1).

**Table 1:** Laboratory test results of important parameters at diagnosis and in January 2024.

Parameter	At diagnosis (12/2022)	January 2024	Reference range
Baseline cortisol	88 nmol/L (3.19 µg/dL)	168 nmol/L (6.1 µg/dL)	133–537 nmol/L (4.8–19.4 µg/dL)
Cortisol after ACTH stimulation		170 nmol/L (6.2 µg/dL)	Cut-off >500 nmol/L (18.1 µg/dL)
ACTH	429 pmol/L (1949 ng/L)		<13.4 pmol/L (<61 ng/L)
Renin	3.15 pmol/L (222.4 mU/L)	0.27 pmol/L (18.8 mU/L)	0.04–0.57 pmol/L (2.8–39.9 mU/L (lying position))
Potassium	5.9 mmol/L	4.3 mmol/L	3.4–4.5 mmol/L
Sodium	134 mmol/L	140 nmol/L	136–145 mmol/L
Creatinine	200 µmol/L (2.273 mg/dL)	106 µmol/L	44–80 µmol/L (0.5–0.909 mg/dL)

Blood count analysis at the time of diagnosis showed platelets of 252 × 10^9^/L. Nevertheless, during the weeks prior to diagnosis, the level of platelet counts undulated between 9 and 400 × 10^9^/L (normal range 143–400 × 10^9^/L), while the INR was between 2.0 and 5.3.

The physical examination at the time of diagnosis showed hyperpigmentation of the lips and oral mucosa.

## Treatment

Based on the laboratory and radiological findings, as well as the physical examination, a diagnosis of adrenal crisis due to the destruction of both adrenal glands based on hemorrhage was made. A substitution with hydrocortisone was initiated immediately. The initial dose of hydrocortisone was a cumulative 50 mg/ day, which was divided into 30 mg in the morning and 20 mg at midday to mimic the natural course of cortisol. Phenprocoumon was temporarily paused. The patient refused hospitalization.

## Outcome and follow-up

We initially saw the patient every few weeks in our outpatient clinic for follow-up checks. The hydrocortisone substitution led to a rapid improvement in symptoms and restoration of well-being, so that we could gradually reduce the dose of hydrocortisone to a maintenance dose of 20–10–0 mg/ day. The patient is affected by obesity, with a body mass index of 40.8 kg/m^2^, which is why we chose an initial maintenance dose of cumulative 30 mg/day, while a reduction to a cumulative dose of 25 mg/day is planned. As an alternative to a twice-daily dose, hydrocortisone can also be prescribed three times a day.

Hydrocortisone has a weak mineralocorticoid effect. For doses of less than 50 mg of hydrocortisone/ day, we added fludrocortisone 0.05 mg/ day to maintain normotension, as well as normonatremia and normokalemia. At her last presentation at our outpatient clinic (January 2024), the levels of sodium and potassium were well balanced.

Follow-up examinations of endogenous cortisol levels and a high-dose (250 μg) short ACTH (1–24) test indicated persistent hypocortisolism after the short-term discontinuation of hydrocortisone (Table 1).

A medical alert notification and a steroid emergency card were given to the patient at the time of diagnosis. Furthermore, we instructed the patient to adjust the dose of hydrocortisone in cases of illness, trauma, or stressful events during every follow-up check in our outpatient clinic. It is crucial that the patient is well informed to avoid adrenal crisis.

## Discussion

As mentioned above, in about 80% of cases, primary adrenocortical insufficiency is caused by autoimmune adrenalitis (1). Compared to that, adrenal hemorrhage is a rare condition that can lead to adrenocortical insufficiency if it occurs bilaterally. Adrenal hemorrhage can cause abdominal pain, which is often misinterpreted by clinicians. In retrospect, the abdominal pain episode in November could be related to the bilateral hemorrhage, which would explain why the MRI did not reveal any fresh hemorrhage. Furthermore, symptoms of adrenocortical insufficiency such as nausea, vomiting, anorexia, weight loss, hypotension, and fatigue, are nonspecific, which makes the diagnosis more difficult (1, 2). In the presented case, abdominal symptoms due to adrenocortical insufficiency were misinterpreted as a urinary tract infection and gastritis.

Given the non-specific symptoms of adrenal hemorrhage, imaging is an important diagnostic tool. CT imaging is usually sufficient for diagnosis. However, in some patients, CT may not be able to diagnose adrenal hemorrhage, and an MRI needs to be added. Compared to CT scans, MRI is the more accurate imaging modality. On the other hand, CT scans are more time-efficient and are recommended as the first imaging tool (7, 8). In addition, a proper physical examination and history-taking play a crucial role in the diagnosis. A more detailed physical examination, noticing of the typical hyperpigmentation of the oral mucosa, might have led to an earlier diagnosis.

Bilateral adrenal hemorrhage has been described in patients on phenprocoumon as well as novel oral anticoagulants (7, 9). Our patient showed INR values up to 5.3, indicating overdosing and consecutively increasing the risk of bleeding. Other risk factors that contribute to an increased risk of bleeding are hematologic diseases such as myelodysplastic syndrome and chronic ITP. Among patients with chronic ITP, the 1-year risks were 1.2% for venous thromboembolism and 7.5% for bleeding (5). Transferred to our patient, besides anticoagulation, the chronic ITP was another independent risk factor, which was active during the weeks prior to diagnosis, with low platelet counts of a minimum of 9 × 10^9^/L.

Case reports exist for adrenal hemorrhages in hematologic patients, e.g. with antiphospholipid syndrome or heparin-induced thrombocytopenia. Bilateral adrenal hemorrhage is related to the prothrombotic state of those diseases. The underlying mechanism is supposed to be an adrenal vein thrombosis followed by hemorrhagic infarction of the adrenal glands. Due to the vascular anatomy with limited venous drainage, the adrenal glands are predisposed to thrombosis (10, 11). At the time of diagnosis, our patient was receiving eltrombopag, which has prothrombogenic effects. Thus, our patient had risk factors for bleeding (thrombocytopenia, anticoagulation) as well as for thrombosis (eltrombopag). The mechanism by which the adrenal hemorrhage occurred could therefore be the same as in antiphospholipid syndrome or heparin-induced thrombocytopenia.

Adrenal dysfunction is mostly irreversible. Nevertheless, adrenocortical function may recover in rare cases, at least partially. To test adrenocortical function, the short high-dose (250 μg) ACTH (1–24) test is considered as the gold standard. During this test, 250 μg of synthetic ACTH (ACTH (1–24) is administered intravenously. To determine adrenal response to exogenous ACTH, serum cortisol levels are measured 30 or 60 min after administration. Peak cortisol levels <500 nmol/L (<18.1 μg/dL) are suggestive of adrenal insufficiency (12).

Our patient has an ongoing disruption of the adrenocortical function; therefore, hydrocortisone and fludrocortisone substitution is still necessary. More than 1 year after the initial diagnosis, recovery of function now seems unlikely.

In conclusion, adrenal hemorrhage is a rare cause of adrenocortical insufficiency and can be easily overlooked due to its non-specific symptoms. It can lead to life-threatening hemodynamic consequences; thus, it should be taken into account, especially in patients with risk factors for bleeding.

## Declaration of interest

The authors declare that there is no conflict of interest that could be perceived as prejudicing the impartiality of the case study reported.

## Funding

This work did not receive any specific grant from any funding agency in the public, commercial, or not-for-profit sector.

## Patient consent

Written informed consent for publication of their clinical details and clinical images was obtained from the patient.

## Author contribution statement

All authors made individual contributions to authorship. SH was involved in the diagnosis and management of this patient and manuscript submission. SH and FB were involved in manuscript draft. All authors reviewed and approved the final draft.

## References

[1] Barthel A Benker G Berens K Diederich S Manfras B Gruber M Kanczkowski W Kline G Kamvissi-Lorenz V Hahner S, *et al.* An update on Addison’s disease. Experimental and Clinical Endocrinology and Diabetes 2019 127 165–175. (10.1055/a-0804-2715) 30562824

[2] Husebye ES Pearce SH Krone NP & Kämpe O. Adrenal insufficiency. Lancet 2021 397 613–629. (10.1016/S0140-6736(21)00136-7) 33484633

[3] Hahner S Spinnler C Fassnacht M Burger-Stritt S Lang K Milovanovic D Beuschlein F Willenberg HS Quinkler M & Allolio B. High incidence of adrenal crisis in educated patients with chronic adrenal insufficiency: a prospective study. Journal of Clinical Endocrinology and Metabolism 2015 100 407–416. (10.1210/jc.2014-3191) 25419882

[4] Cooper N & Ghanima W. Immune thrombocytopenia. New England Journal of Medicine 2019 381 945–955. (10.1056/NEJMcp1810479) 31483965

[5] Adelborg K Kristensen NR Nørgaard M Bahmanyar S Ghanima W Kilpatrick K Frederiksen H Ekstrand C Sørensen HT & Fynbo Christiansen C. Cardiovascular and bleeding outcomes in a population-based cohort of patients with chronic immune thrombocytopenia. Journal of Thrombosis and Haemostasis 2019 17 912–924. (10.1111/jth.14446) 30933417

[6] Schoonen WM Kucera G Coalson J Li L Rutstein M Mowat F Fryzek J & Kaye JA. Epidemiology of immune thrombocytopenic purpura in the general practice research database. British Journal of Haematology 2009 145 235–244. (10.1111/j.1365-2141.2009.07615.x) 19245432

[7] Alidoost M Soomro R Gubeladze A Morabia A Holland S Asif A & Hossain MA. Rivaroxaban related bilateral adrenal hemorrhage: a rare complications of direct oral anticoagulants - a case reports. American Journal of Case Reports 2019 20 1607–1611. (10.12659/AJCR.917780) 31676747 PMC6849691

[8] Di Serafino M Severino R Coppola V Gioioso M Rocca R Lisanti F & Scarano E. Nontraumatic adrenal hemorrhage: the adrenal stress. Radiolology Case Reports 2017 12 483–487. (10.1016/j.radcr.2017.03.020) PMC5551907 28828107

[9] Park KJ Bushmiaer M & Barnes CL. Bilateral adrenal hemorrhage in a total knee patient associated with enoxaparin usage. Arthroplasty Today 2015 1 65–68. (10.1016/j.artd.2015.02.001) 28326373 PMC4956683

[10] Rosenberger LH Smith PW Sawyer RG Hanks JB Adams RB & Hedrick TL. Bilateral adrenal hemorrhage: the unrecognized cause of hemodynamic collapse associated with heparin-induced thrombocytopenia. Critical Care Medicine 2011 39 833–838. (10.1097/CCM.0b013e318206d0eb) 21242799 PMC3101312

[11] Ramon I Mathian A Bachelot A Hervier B Haroche J Boutin-Le Thi D Costedoat-Chalumeau N Wechsler B Karmali R Velkeniers B, *et al.* Primary adrenal insufficiency due to bilateral adrenal hemorrhage-adrenal infarction in the antiphospholipid syndrome: long-term outcome of 16 patients. Journal of Clinical Endocrinology and Metabolism 2013 98 3179–3189. (10.1210/jc.2012-4300) 23783099

[12] Bornstein SR Allolio B Arlt W Barthel A Don-Wauchope A Hammer GD Husebye ES Merke DP Murad MH Stratakis CA, *et al.* Diagnosis and treatment of primary adrenal insufficiency: an Endocrine Society clinical practice guideline. Journal of Clinical Endocrinology and Metabolism 2016 101 364–389. (10.1210/jc.2015-1710) 26760044 PMC4880116

